# Latent profiles of AI literacy among K-12 students: predictors and links to self-regulated learning

**DOI:** 10.3389/fpsyg.2026.1834851

**Published:** 2026-06-05

**Authors:** Dan Wang, Qi Wang, Shan Zhang

**Affiliations:** 1 School of Physical Education, Hubei University of Education, Wuhan, Hubei, China; 2 Faculty of Artificial Intelligence Education, Central China Normal University, Wuhan, Hubei, China

**Keywords:** artificial intelligence literacy, family socioeconomic status, K-12 education, latent profile analysis, self-regulated learning

## Abstract

**Introduction:**

Artificial intelligence (AI) literacy has become increasingly critical as AI integrates into K-12 education, yet understanding of the factors influencing students' AI literacy and its relationship with self-regulated learning (SRL) remains limited.

**Methods:**

To address this gap, the present study draws on large-scale survey data from 11,020 Chinese K-12 students and employs latent profile analysis (LPA) to identify distinct AI literacy profiles and to systematically examine their predictors and associations with SRL.

**Results:**

The results revealed four qualitatively distinct and progressively ordered AI literacy profiles: the foundational-limited profile, the moderate-stable profile, the advanced-developing profile, and the high-excellence profile. Multinomial logistic regression analyses indicated that students' gender, only-child status, educational stage, family socioeconomic status, frequency of AI use, parental active mediation, and school AI support significantly predicted profile membership. Further analyses showed significant differences in SRL across the identified profiles, with students in higher AI literacy profiles demonstrating consistently stronger SRL abilities.

**Discussion:**

These findings provide empirical evidence to support the development of inclusive and differentiated AI literacy education in K-12 settings and offer important implications for fostering students' SRL in AI-enhanced learning environments.

## 1 Introduction

The emergence and widespread accessibility of generative artificial intelligence (AI) technologies, exemplified by ChatGPT, have significantly accelerated the diffusion of powerful AI applications. This rapid proliferation underscores the need for future generations to develop adequate AI literacy to engage with these technologies effectively, critically, and ethically (Long and Magerko, 2020; Zhong and Liu, 2025). For K-12 students, cultivating AI literacy from an early age not only equips students with the knowledge and skills needed to address complex problems in an AI-driven society but also motivates students from disadvantaged groups to pursue further study or careers in the AI field (Ng et al., 2024a,b). Consequently, many countries have begun integrating AI education into their basic education systems (Miao and Shiohira, 2022; Touretzky et al., 2023).

Despite this urgent need, research and practice on AI literacy at the K-12 level remain limited (Casal-Otero et al., 2023; Yim and Su, 2025), which limits the evidence we have for creating specific interventions. On the one hand, significant differences exist among students in understanding, engaging with, and applying AI-related knowledge (Dong et al., 2025), suggesting the potential presence of distinct learner profiles. However, most studies still treat all students the same, ignoring differences among them and making instructional design less precise. On the other hand, existing research on predictors of AI literacy has focused largely on university students and teachers (Gu and Ericson, 2025), leaving a gap in empirical evidence regarding K-12 learners. Moreover, while factors such as family background, school support, and frequency of use are known to influence technological literacy (Loh et al., 2025; Ren et al., 2022), how these multilevel factors collectively shape different types of AI literacy among K-12 students remains an under-explored and poorly integrated issue. These gaps highlight the need to investigate the underlying patterns of K-12 students' AI literacy and their influencing factors.

In parallel, the application of AI in K-12 education has grown rapidly (Yim and Su, 2025), with keen academic interest in leveraging AI—such as chatbots, adaptive feedback systems, and intelligent tutoring systems—to support and enhance students' self-regulated learning (SRL) (Zhang and Derakhshan, 2025; Zhang and Wang, 2025). Broadly defined, SRL is an active, constructive process whereby learners set goals, monitor their progress, and adjust their strategies to achieve desired learning outcomes (Zimmerman, 2002). Critically, AI literacy, defined as the ability to critically evaluate and ethically use AI (Ng et al., 2021), provides the essential agency required to navigate these AI-enhanced environments. Without such literacy, students risk delegating their learning responsibilities to technology, potentially succumbing to over-reliance (Zhang and Chen, 2025). However, this body of work has primarily centered on tool design and intervention efficacy, paying scant attention to how variations in students' own AI literacy might influence SRL outcomes. This oversight is problematic, as human-AI collaborative learning carries risks such as cognitive rigidity, over-reliance, and the delegation of learning responsibility (Li et al., 2024). Therefore, focusing solely on the capabilities of AI tools, without considering learners' understanding and agentic use of AI, may lead to inflated expectations of AI's benefits for SRL and potentially obscure educational inequalities (Chen and Delaney, 2025).

Given the significance of these issues and the limitations of existing research, the present study addresses the following research questions:

RQ1. What distinct AI literacy profiles can be identified among K-12 students?

RQ2. How do individual, family, and school factors predict students' membership in different AI literacy profiles?

RQ3. How does SRL differ across the identified AI literacy profiles?

Our findings offer insights to inform instructional practices and intervention strategies that enhance students' AI literacy and SRL, thereby promoting inclusiveness and diversity in K-12 AI education (Xia et al., 2022).

## 2 Literature review

### 2.1 AI literacy

As a higher-order form of digital literacy (Yang, 2022), AI literacy has attracted increasing attention in recent educational research (Casal-Otero et al., 2023; Ng et al., 2024a,b). With the growing integration of AI in education, its conceptual scope has expanded. Scholars have defined AI literacy as the ability to critically evaluate and effectively apply AI technologies (Long and Magerko, 2020), encompassing areas such as AI concepts, processes, interaction, and societal implications (Touretzky et al., 2019). Ng et al. (2021) proposed a widely adopted four-dimensional framework—knowing and understanding AI, using and applying AI, evaluating and creating AI, and AI ethics—later echoed by similar models (Wang et al., 2023). Despite definitional variations, most frameworks align with Bloom's taxonomy (Ng et al., 2021). Accordingly, this study adopted Ng et al. (2021)'s four-domain framework, emphasizing K-12 students as active, critical, and responsible participants in AI-related learning and practice.

The proliferation of generative artificial intelligence tools, such as ChatGPT and Sora, has fostered a broad consensus on the necessity of implementing AI literacy education within K-12 settings (Chiu, 2024; Pedro et al., 2019; Wang and Lester, 2023). Concurrently, research in K-12 AI education has shifted from asking whether AI should be taught to exploring how it can be taught effectively. An expanding body of work has begun investigating pedagogical strategies and tools designed to scaffold AI literacy for young learners (Yim and Su, 2025). Despite growing scholarly interest in integrating AI into school curricula, K-12 research still lacks clear guidance on developmentally appropriate AI literacy content and pedagogy (Su et al., 2024). Recent empirical evidence reveals substantial variation in how students understand and interact with AI (Dong et al., 2025), suggesting that learners are not a homogeneous group but rather possess distinct AI literacy profiles. Furthermore, while researchers like Zhang and Kang (2026) and Sun and Chan (2025) have utilized latent profile analysis to identify latent types of AI literacy among middle school and university students, research specifically targeting the broader K-12 student population remains limited. This leaves a significant gap in our understanding of early AI literacy development patterns among children and adolescents. Therefore, it is essential to utilize latent profile analysis to examine the diverse AI literacy types among K-12 students. Latent profile analysis is a person-centered approach that, unlike variable-centered methods that examine average relationships within a sample, captures heterogeneity within a population by grouping individuals with similar characteristics (Howard and Hoffman, 2018). This method is particularly suited for informing the design of differentiated and targeted instructional support in K-12 AI literacy education, as it enables educators to tailor interventions to the specific needs of different learner subgroups.

### 2.2 Factors affecting AI literacy

AI literacy education is a complex phenomenon, making it critical to analyze the factors shaping AI literacy from multiple perspectives (Feher and Katona, 2021). In line with this, Celik (2023) explores potential factors influencing AI literacy by integrating individual and socio-cultural perspectives, offering a unified understanding and an approach that informs the present study. In this study, we focused on several demographic and sociological variables, including gender, only-child status, educational stage, family socioeconomic status (SES), frequency of AI use, parental active mediation, and school AI support.

Research on gender differences in AI literacy remains inconclusive. Studies at the university level show mixed patterns: some report higher AI literacy among males (Toker Gokce et al., 2025), while others find no gender gap in knowledge or attitudes (Kovačević and Demić, 2024). Notably, Jang et al. (2022) reported that females demonstrated stronger awareness of AI ethics. Similar inconsistencies appear in K-12 studies. Zhong and Liu (2025) found that male secondary students showed higher AI knowledge, but females reported stronger affective engagement. Dai et al. (2020) identified higher AI literacy and confidence among male upper-elementary students, whereas Kim and Kwon (2024) observed no gender differences in most AI literacy indicators among sixth graders, aside from males' better performance in machine learning tasks. Overall, existing evidence suggests gender effects are context-dependent and vary across dimensions of AI literacy.

Students' academic stage is a key factor, with notable differences in cognitive development and abstract thinking across ages. Research indicates that primary school students typically engage with AI through contextualized experiences, while secondary students begin to grasp its underlying logic and mechanisms (Su and Yang, 2024). Additionally, although direct evidence on only-child status and AI literacy is limited internationally, the resource dilution hypothesis suggests that only children may receive more concentrated parental resources, enhancing their access to educational electronic devices and early learning tools (Wang and Feng, 2021).

Frequency and manner of exposure to AI tools constitute direct influences on the development of AI literacy. Faruqe et al. (2021) posit that AI literacy competencies are contingent upon the frequency and intensity of interaction with AI technologies, suggesting that individuals with less frequent exposure tend to demonstrate lower levels of AI literacy. Corroborating this, recent empirical studies have likewise suggested that the frequency with which individuals use AI technologies may play an important role in shaping their AI literacy (Seker et al., 2025; Toker Gokce et al., 2025).

Family SES, which encompasses parental education, income, and occupation, significantly shapes how individuals understand and use technology (Bradley and Corwyn, 2002). Research consistently shows that higher family SES is linked to greater digital literacy (Ren et al., 2022; Van Deursen and Van Dijk, 2014). Notably, studies by Zhang et al. (2024) found a significant positive correlation between family SES and students' AI literacy, noting that this association is even stronger than the relationship between family SES and general digital literacy. These findings suggest that family background may play an even more pivotal role in the acquisition of AI-related knowledge and competencies than it does in the development of traditional digital skills. In addition, parental digital mediation, including active guidance, restrictive control, and co-use, significantly influences children's digital competencies. Active mediation is positively associated with adolescents' digital skills (Cabello-Hutt et al., 2018) and can fully mediate the effect of family SES on digital maturity (Koch et al., 2024; Loh et al., 2025), indicating that both family SES and parental mediation may serve as key factors shaping students' AI literacy.

Schools are instrumental in advancing students' AI literacy, offering a foundational support system that includes relevant courses, organized competitions, and the provision of qualified teaching staff and laboratory resources (Loh et al., 2025). However, it is noteworthy that some studies have reported a negative predictive effect of school environment and support on students' AI literacy (Luo et al., 2025). This finding diverges from conclusions in existing information literacy research. For example, Martin (2011) argued that structured school support, such as curriculum-integrated instruction, contributes to the improvement of students' information literacy; similarly, Wu et al. (2022) emphasized the central role that schools play in fostering students' information literacy development. This discrepancy also provides a valuable point of departure for the present study to further examine why school support may exert divergent effects on students' AI literacy.

### 2.3 The relationship between AI literacy and self-regulated learning

SRL refers to learners' ability to actively set goals, monitor progress, and adjust strategies to achieve desired learning outcomes (Zimmerman, 2002). Students with strong SRL skills not only demonstrate greater persistence and stress management in learning (Namaziandost et al., 2023) but also effectively leverage external support—such as teachers, peers, or technological tools—when dealing with complex or unfamiliar learning tasks (Xia et al., 2023). However, traditional forms of external support often involve supervision and are constrained by time and space (Boekaerts, 1999), making it difficult to provide sustained and personalized assistance.

With the rapid proliferation of AI, particularly generative AI, AI tools are increasingly used to support students' SRL processes. Empirical studies show that AI applications such as chatbots, adaptive feedback systems, and intelligent tutoring systems can provide personalized support during the forethought, performance, and reflection phases of SRL (Ng et al., 2024c; Wang and Lin, 2023) For example, the SRLbot developed by Ng et al. (2024b) offers dynamic learning suggestions based on students' real-time learning states, while study demonstrates that ChatGPT can significantly enhance learners' planning and self-monitoring during the reflection stage (Fan et al., 2025).

Despite these promising findings, existing research predominantly focuses on the functions or effects of AI tools themselves (Järvelä et al., 2023), while paying limited attention to learners' differences in AI literacy. This oversight introduces two potential risks. First, students with different levels of AI literacy may use AI in fundamentally different, even opposite, ways (Yang and Zhao, 2024), which can lead to divergent or even negative learning outcomes when using the same tool. Second, insufficient ability to critically evaluate AI may cause students to rely excessively on AI systems, weakening their autonomy and inducing cognitive inertia (Fan et al., 2025; Li et al., 2024). Therefore, focusing solely on what AI tools can do, without considering learners' understanding and use of AI, may result in overly optimistic expectations of AI's impact on SRL and obscure potential educational inequalities (Chen and Delaney, 2025).

A small but growing body of research has begun to examine the direct relationship between AI literacy and SRL. Ng et al. (2021) emphasized in their review that AI literacy involves not only technical operation skills but also the abilities to critically evaluate and ethically use AI—dimensions closely associated with SRL sub-processes such as goal setting, monitoring, and strategy adjustment. Furthermore, Zhang and Chen (2025) identified four learner profiles with different patterns in a survey of 1,704 Chinese university students. Their findings revealed that “AI-Inclined” learners, despite having relatively high AI literacy, tended to over-rely on AI due to weaker SRL, whereas “Potential” learners displayed a mutually reinforcing developmental pattern between AI literacy and SRL. These results highlight that differences in AI literacy are crucial for understanding students' SRL performance.

## 3 Methods

### 3.1 Participants

The data were collected through the Questionnaire on the Current Status of Digitalization in Primary and Secondary Schools (Student Version), administered by the research team between March and June 2025. The questionnaire covered students' demographic characteristics, family background, and experiences with AI education.

A stratified sampling strategy was used. Survey sites were selected from eastern (Guangdong, Zhejiang), central (Hubei, Hunan, Jiangxi), and western (Guizhou, Sichuan) China, with on-site surveys conducted in 21 counties or districts across seven provinces. Prior to data collection, informed consent was obtained from schools and students, and anonymity and confidentiality were ensured.

After data screening, questionnaires with more than 30% missing data, obvious response patterns, or failed attention checks were excluded. The final sample comprised 11,020 valid responses, yielding an effective response rate of over 90%.

### 3.2 Measures

To ensure the linguistic equivalence and cultural relevance of the instruments within the Chinese context, a rigorous translation and back-translation procedure was implemented (Brislin, 1980). Initially, the original English items were translated into Chinese by two researchers specializing in Educational Technology. Subsequently, a bilingual scholar, who was blinded to the original scales, performed a back-translation to ensure semantic consistency. To further evaluate cultural applicability, a pilot study was conducted among students from a primary and a secondary school (*N* = 263). Based on the pilot results, item phrasing was refined for clarity, and preliminary reliability and validity analyses were performed. Following these analyses, items with poor psychometric performance were removed. The final modified scales demonstrated robust internal consistency and structural validity, confirming their suitability for measuring AI literacy and its predictors among Chinese schoolchildren.

#### 3.2.1 Artificial intelligence literacy

The AI literacy scale was adapted from Ng et al. (2024a) and consists of 22 items across four subscales: Know and understand (5 items), Use and apply (4 items), Evaluate and create (4 items), and AI ethics (9 items). The second-order model demonstrated good fit indices (CFI = 0.994, TLI = 0.981, SRMR = 0.04, RMSEA = 0.059). Participants responded to each item on a 5-point Likert scale ranging from 1 (strongly disagree) to 5 (strongly agree).

#### 3.2.2 Self-regulated learning

SRL was measured using a scale adapted from (Hong et al., 2015). The scale includes three dimensions, each assessed with three items: goal setting (e.g., “I try to analyze my weaknesses across different courses and set goals to address them”; α = 0.832), performance monitoring (e.g., “I always check whether my studying approaches meet the need of improving test performance”; α = 0.865), and pursuing goals (e.g., “To achieve the learning goal, I will adopt a new learning approach to study”; α = 0.894). All items were rated on a 5-point Likert scale (1 = strongly disagree, 5 = strongly agree). The second-order model demonstrated good fit indices (CFI = 0.978, TLI = 0.969, SRMR = 0.02, RMSEA = 0.064).

#### 3.2.3 Family socioeconomic status

Family SES was assessed using parental education, parental occupation, and family wealth. Parental education was recoded into approximate years of schooling (0–19) based on the highest level attained by either parent. Parental occupation was scored on a 1–9 scale, again using the higher parental score. Family wealth was measured using household asset indicators adapted from PISA 2018. The three standardized indicators were combined using principal component analysis (PCA) to generate a composite family SES score, with higher values indicating greater socioeconomic advantage (Chung et al., 2017; Fraillon et al., 2020).

#### 3.2.4 Parental active mediation

Parental active mediation was measured using an adapted version of the parental mediation scale developed by Nikken and Jansz (2014). The revised scale consists of five items, such as “My parents tell me how to use different types of smart devices” and “My parents guide me to explore the resources and functions of smart devices.” All items were rated on a five-point Likert scale, with response options ranging from 1 (“never”) to 5(“always”). The scale demonstrated high internal consistency (Cronbach's α = 0.876) and good construct validity (CFI = 0.913, TLI = 0.946, SRMR = 0.04, RMSEA = 0.042).

#### 3.2.5 Frequency of AI use

Students' frequency of AI use was assessed through multiple items measuring how often they used various AI devices at home, including smartphones, smart speakers, smart learning tablets/educational devices, smartwatches, translation pens, and generative AI applications (e.g., Doubao, Kimi, DeepSeek). Items were scored on a 4-point scale, with 1 representing “almost never,” 2 “occasionally,” 3 “often,” and 4 “always.” The scale demonstrated high internal consistency (Cronbach's α = 0.931).

#### 3.2.6 School AI support

School AI support was measured using a scale adapted from Louis et al. (1996). The revised scale includes four items capturing schools' support for AI-related infrastructure, access to resources, and training opportunities (e.g., “My school provides sufficient resources for learning about artificial intelligence,” “My school offers training courses on how to use AI tools for learning”). Items were rated on a 5-point Likert scale ranging from 1 (strongly disagree) to 5 (strongly agree). The scale exhibited high reliability (Cronbach' s α = 0.873) and good model fit (CFI = 0.922, TLI = 0.957, SRMR = 0.04, RMSEA = 0.054).

### 3.3 Data analysis strategy

To address Research Question 1, LPA was conducted in Mplus 8.10 to identify distinct AI literacy profiles among K-12 students. Models with one to five profiles were estimated and compared using multiple fit indices, including AIC, BIC, ABIC, entropy, the Lo-Mendell-Rubin (LMR) test, and the bootstrap likelihood ratio test (BLRT) (Marsh et al., 2009; Tein et al., 2013). Better model fit was indicated by lower information criteria, higher entropy (≥0.80), and significant LMR/BLRT results (*p* < 0.05). Profiles comprising less than 5% of the sample were excluded (Bauer, 2022). The final model was selected based on statistical fit, parsimony, and theoretical interpretability.

To address Research Question 2, gender, only-child status, school level, family SES, AI use frequency, parental active mediation, and school AI support were included as predictors of AI literacy profile membership using the three-step R3STEP procedure in Mplus (Asparouhov and Muthén, 2014a). This approach applies multinomial logistic regression while accounting for classification error and preserving the latent profile structure; odds ratios were reported. The MLR estimator was used to obtain robust standard errors.

To address Research Question 3, differences in SRL across AI literacy profiles were examined using the BCH method (Asparouhov and Muthén, 2014b), which accounts for classification uncertainty in continuous distal outcomes. Overall group differences were tested using Wald χ^2^ statistics (*p* < 0.05), followed by pairwise comparisons when significant.

## 4 Results

### 4.1 Preliminary analysis

Table 1 presents the descriptive statistics and Pearson correlations among the study variables. All variables were significantly and positively correlated. AI literacy showed the strongest association with SRL (*r* = 0.411, *p* < 0.001), followed by parental active mediation (*r* = 0.265, *p* < 0.001) and school AI support (*r* = 0.253, *p* < 0.001). Family SES was modestly related to AI use frequency and AI literacy.

**Table 1:** Descriptive statistics and correlations for key variables.

Variables	1	2	3	4	5	6	Mean	SD
1. Family socioeconomic status	1						0	1
2. Parental active mediation	0.153^***^	1					3.293	1.011
3. School AI support	0.059^***^	0.184^***^	1				2.742	0.927
4. Frequency of AI use	0.239^***^	0.155^***^	0.070^***^	1			1.607	0.414
5. Self-regulated learning	0.130^***^	0.336^***^	0.220^***^	0.134^***^	1		3.323	0.855
6. AI literacy	0.183^***^	0.265^***^	0.253^***^	0.214^***^	0.411^***^	1	3.164	0.757

^***^p < 0.001.

### 4.2 RQ1: identifying profiles of students' AI literacy

**Figure 1:**
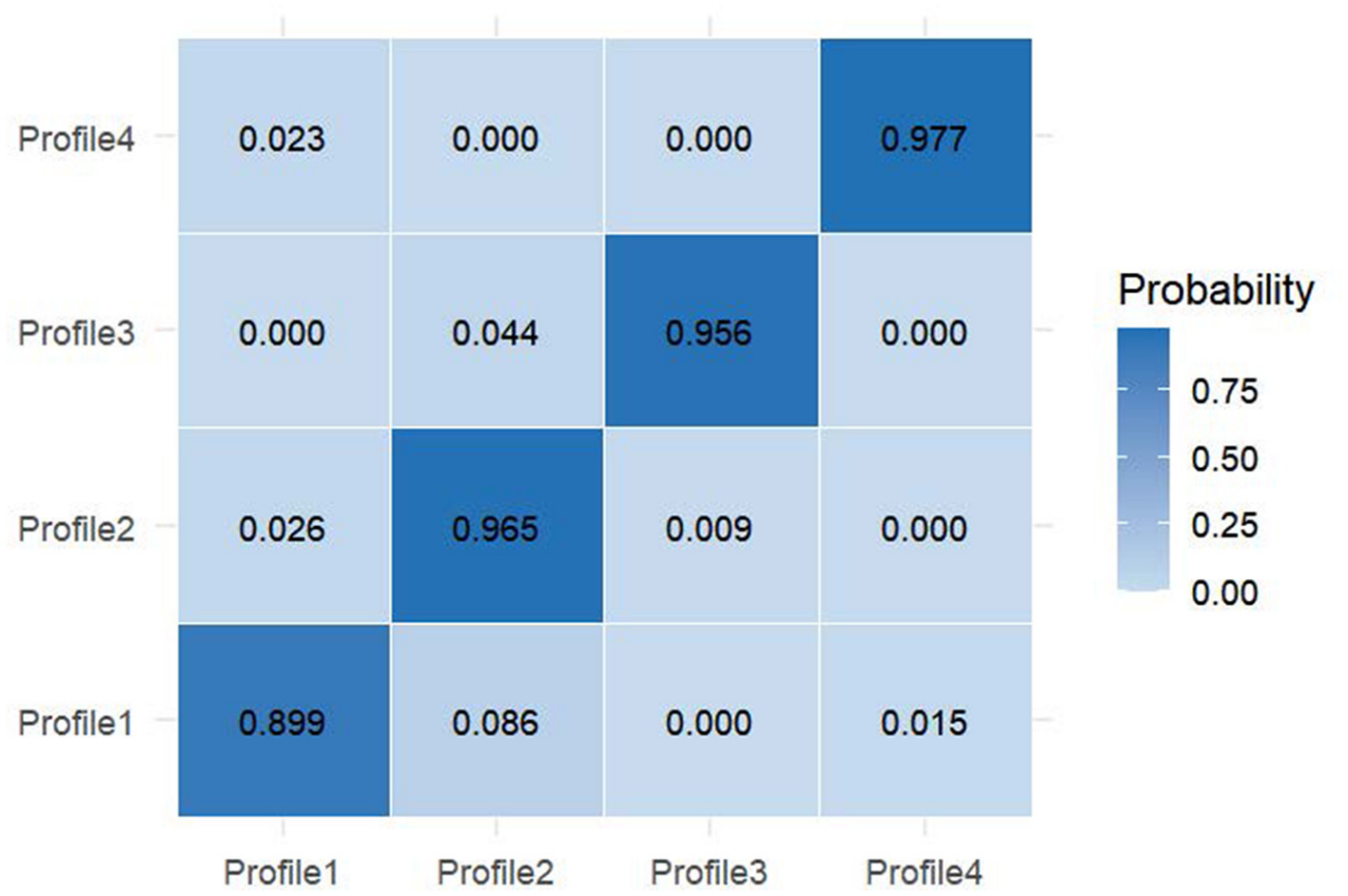
Average latent class probabilities for most likely latent class membership by latent class.

To address RQ1, Latent Profile Analysis (LPA) was conducted using composite scores of the four AI literacy dimensions. Models with one to five profiles were compared (see Table 2). Information criteria (AIC, BIC, aBIC) decreased as the number of profiles increased, indicating improved fit, and both BLRT and LMR tests supported solutions with more than one profile. Although the five-profile model yielded the lowest information criteria, it included a very small class (4.2%), raising concerns about stability and interpretability. The four-profile solution demonstrated excellent classification accuracy (Entropy = 0.949), acceptable class sizes (smallest = 6.6%), and clear substantive differentiation. Therefore, balancing model fit, parsimony, and theoretical interpretability, the four-profile solution was selected for subsequent analyses. Figure 1 illustrates that the high average posterior probabilities (ranging from 89.9% to 97.7% for Profiles 1–4) further substantiate the robustness of this four-profile solution.

**Table 2:** Model fit indices for different profile solutions.

Model	Loglikelihood	AIC	BIC	ABIC	Entropy	BLRT	LMR	Profile size
1	−59163.201	111512.238	111570.40	111544.977	–	–	–	1
2	−53478.184	100959.539	101054.053	101012.741	0.904	0	0	0.837/0.162
3	−47028.857	89279.527	89410.393	89353.191	0.939	0	0	0.110/0.747/0.142
4	−45301.342	86118.096	86285.313	86212.223	0.949	0	0	0.106/0.065/0.143/0.684
5	−42669.378	81223.787	81427.355	81338.375	0.920	0	0	0.042/0.166/0.139/0.584/0.067

**Table 3:** Comparison results of AI literacy across latent profiles.

AI literacy dimension	Profile 1 (*n* = 1174)	Profile 2 (*n* = 7540)	Profile 3 (*n* = 1584)	Profile 4 (*n* = 722)	*F*	*Post-hoc* test
KU	1.73 ± 0.67	3.06 ± 0.43	4.01 ± 0.48	4.90 ± 0.29	12256.05^***^	4> 3> 2> 1
UA	1.47 ± 0.49	2.91 ± 0.37	3.83 ± 0.36	4.92 ± 0.20	19785.89^***^	4> 3> 2> 1
EC	1.39 ± 0.54	2.78 ± 0.56	3.59 ± 0.61	4.87 ± 0.35	25256.01^***^	4> 3> 2> 1
AE	2.47 ± 1.27	3.40 ± 0.71	4.10 ± 0.61	4.83 ± 0.48	17568.21^***^	4> 3> 2> 1

^***^p < 0.001. KU, know and understand; UA, use and apply; EC, evaluate and create; AE, AI ethics.

**Table 4:** Multinomial logistic regression results.

Variable	Ref: foundational-limited profile								
	Moderate-stable profile			Advanced-developing profile			High-excellence profile		
	B	SE	OR	B	SE	OR	B	SE	OR
Family socioeconomic status	0.035^*^	0.016	1.036	0.118^***^	0.019	1.126	0.118^***^	0.023	1.125
Parental active mediation	0.288^***^	0.043	1.334	0.593^***^	0.053	1.809	0.863^***^	0.07	2.37
Frequency of AI use	0.731^***^	0.132	2.077	1.269^***^	0.147	3.558	1.571^***^	0.163	4.811
School AI support	0.745^***^	0.048	2.107	0.98^***^	0.059	2.664	1.069^***^	0.07	2.913
Single child^a^	−0.205	0.12	0.815	−0.384^**^	0.139	0.681	−0.383^*^	0.164	0.682
Gender^a^	0.089	0.085	1.093	−0.135	0.104	0.874	−0.992^*^	0.137	0.371
Educational stage^a^	−0.451^***^	0.088	0.637	−0.371^***^	0.107	0.69	−0.047	0.135	0.954

^***^p < 0.001; ^**^p < 0.01; ^*^p < 0.05.

^a^ boys, single children, and junior high school were used as the reference groups.

**Table 5:** Self-regulated learning differences across AI literacy profiles.

Variables	Profiles	Mean ±SE	Pairwise comparisons				χ^2^
			C1	C2	C3	C4	
Goal setting	Foundational-limited	2.767 ± 0.037	/				978.334^***^
	Moderate-stable	3.218 ± 0.010	138.452^***^	/			
	Advanced-developing	3.748 ± 0.025	493.950^***^	372.681 ^***^	/		
	High-excellence	4.181 ± 0.047	566.215^***^	407.275^***^	64.633^***^	/	
Pursuing goal	Foundational-limited	2.750 ± 0.035	/				1025.207^***^
	Moderate-stable	3.210 ± 0.010	153.667^***^	/			
	Advanced-developing	3.720 ± 0.024	521.924^***^	369.995^***^	/		
	High-excellence	4.168 ± 0.045	608.330^***^	427.565 ^***^	73.851^***^	/	
Performance monitoring	Foundational-limited	2.754 ± 0.036	/				854.725^***^
	Moderate-stable	3.215 ± 0.010	148.128^***^	/			
	Advanced-developing	3.669 ± 0.025	431.217^***^	254.786^***^	/		
	High-excellence	4.145 ± 0.046	563.568^***^	386.274^***^	78.192^***^	/	
Self-regulated learning	Foundational-limited	2.759 ± 0.036	/				875.989^***^
	Moderate-stable	3.218 ± 0.010	145.699^***^	/			
	Advanced-developing	3.683 ± 0.026	437.028^***^	266.279^***^	/		
	High-excellence	4.164 ± 0.046	571.960^***^	398.289^***^	79.416^***^	/	

^***^p < 0.001. C1, foundational-limited; C2, moderate-stable; C3, advanced-developing; C4, high-excellence.

**Figure 2:**
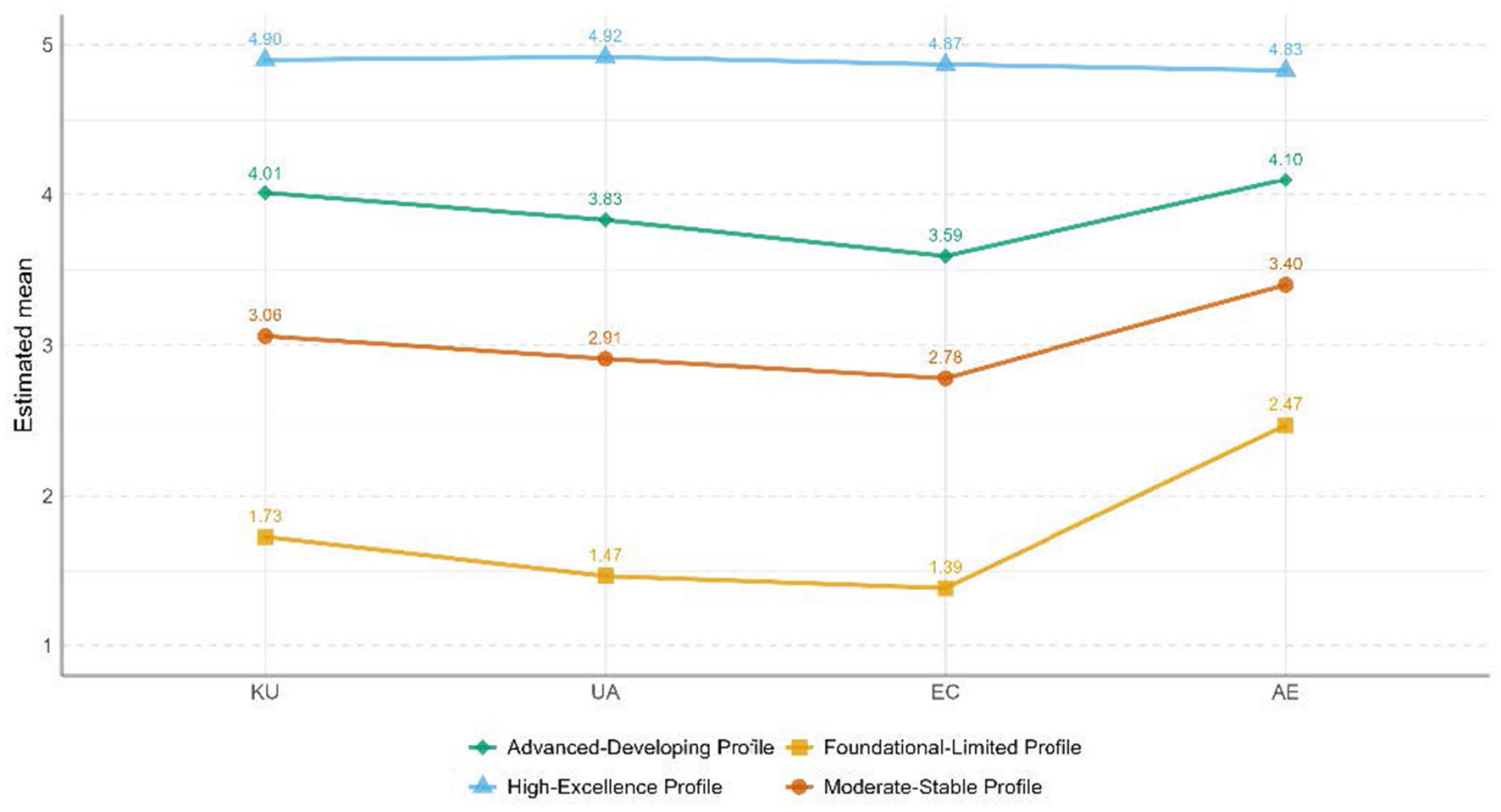
Latent profiles from the four-profile solution with the mean scores.

Figure 2 illustrates the mean scores of the four AI literacy dimensions across the four latent profiles.

Profile 1: Foundational-Limited (*n* = 1,174; 10.65%) exhibited the lowest scores across all dimensions, indicating limited AI knowledge, skills, engagement, and ethics-related understanding.

Profile 2: Moderate-Stable (*n* = 7,540; 68.42%) represented the largest group and showed consistently higher scores than Profile 1, though remaining below the advanced profiles, suggesting basic and stable AI competencies.

Profile 3: Advanced-Developing (*n*=1,584; 14.37%) demonstrated high scores across all dimensions, reflecting strong foundational abilities with clear potential for further growth.

Profile 4: High-Excellence (*n*=722; 6.5%) showed the highest and most consistent scores across all dimensions, indicating exceptional AI knowledge, skills, engagement, and ethical awareness.

Overall, the ANOVA test indicates significant differences in the mean values across the four profiles for every indicator. *Post-hoc* tests revealed a consistent, statistically significant ordinal pattern for all four dimensions: High-Excellence > Advanced-Developing > Moderate-Stable > Foundational-Limited. To visually illustrate these distinct characteristics, the mean scores are plotted in Figure 2.

### 4.3 RQ2: factors predicting AI literacy profile membership

To answer RQ2, based on the final latent profiles, this study employed logistic regression to examine how various factors influence profile membership, using the “Foundational-Limited Profile” as the reference group.

As shown in the table, several factors significantly predicted membership in the higher profiles. Parental active mediation, frequency of AI use, and school AI support were strong, positive predictors across all three non-reference profiles. The Odds Ratios (OR) increased from the Moderate-Stable to the High-Excellence Profile, indicating that higher levels of these factors made students progressively more likely to belong to a more advanced profile. Family SES was also a significant positive predictor for all profiles.

Regarding demographic variables, not being a single child significantly reduced the odds of belonging to the Advanced-Developing (OR = 0.681, *p* < 0.01) and High-Excellence (OR=0.682, *p* < 0.05) profiles. Female students had significantly lower odds of belonging to the High-Excellence Profile (OR=0.371, *p* < 0.001). Furthermore, primary school students were less likely to be in the Moderate-Stable (OR=0.637, *p* < 0.001) and Advanced-Developing (OR = 0.690, *p* < 0.001) profiles compared to junior high school students.

In summary, environmental and behavioral factors like AI use and support were consistently associated with higher probabilities of belonging to more advanced profiles, while certain demographic characteristics also played a significant, though more limited, role.

### 4.4 RQ3: self-regulated learning across AI literacy profiles

Using the BCH chi-square tests, we examined differences in SRL across the four AI literacy profiles. Significant overall differences were found for goal setting (χ^2^ = 978.334, *p* < 0.001), pursuing goals (χ^2^ = 1025.207, *p* < 0.001), performance monitoring (χ^2^ = 854.725, *p* < 0.001), and overall SRL (χ^2^ = 875.989, *p* < 0.001). Across all SRL indicators, mean scores increased steadily from the Foundational-Limited profile to the High-Excellence profile. Students in the Moderate-Stable profile scored significantly higher than those in the Foundational-Limited profile, while the Advanced-Developing profile showed significantly higher scores than both the Foundational-Limited and Moderate-Stable profiles. The High-Excellence group outperformed all other profiles across all SRL dimensions (all *p* < 0.001). Overall, students with more advanced AI literacy profiles consistently demonstrated stronger SRL competencies, indicating a clear positive association between AI literacy level and SRL.

## 5 Discussion

### 5.1 The profiles of K-12 students' AI literacy

This study identified four typical profiles of students' AI literacy: the Foundational-Limited Profile, the Moderate-Stable Profile, the Advanced-Developing Profile, and the High-Excellence Profile. This profile structure aligns with and extends previous research. Specifically, our findings resonate with the work of Sun and Chan (2025), who identified similar competency clusters among secondary school students. Our study generalizes this pattern by incorporating elementary school samples, thereby validating and extending the framework across the entire K-12 continuum. This provides a more comprehensive empirical basis for advocating differentiated AI education strategies tailored to students' varying baseline competencies.

It is noteworthy that the majority of Chinese students fall within the Moderate-Stable Profile, accounting for 68.42% of respondents, whereas only a small proportion (6.3%) belongs to the High-Excellence Profile. This distribution suggests that the overall state of AI literacy among Chinese students may be less advanced than often presumed, highlighting a substantial gap between foundational exposure and genuine high-level proficiency. Given the escalating importance of AI literacy for future citizenship and workforce readiness, this finding underscores an urgent imperative to systematically enhance and deepen AI education initiatives within K-12 curricula (Wang and Lester, 2023).

Across all profiles, we observed that competencies related to AI Knowledge (KU), Application (UA), and AI Ethics generally constituted relatively stronger areas. Conversely, AI literacy in creation and evaluation consistently emerged as a relative weakness, even within the more advanced profiles. This aligns with previous findings in digital literacy research, which suggest that cultivating higher-order cognitive and creative skills is more challenging compared to foundational knowledge and procedural application (Liu et al., 2024; Stéphan et al., 2019), and the same may hold true for K-12 students' AI literacy.

Taken together, the four identified AI literacy profiles reveal a hierarchical, cumulative structure that aligns with the theoretical framing of AI literacy as a competency progressing from foundational knowledge (Know & Understand, Use & Apply) to higher-order cognitive application (Evaluate & Create) (Long and Magerko, 2020; Ng et al., 2021). The consistent ordinal pattern across profiles, with the ‘Evaluate & Create' dimension persistently lagging, suggests that higher-order AI competencies do not automatically emerge from basic skills and thus require explicit instructional design.

### 5.2 The factors of K-12 students' AI literacy

Regarding predictors, environmental and behavioral factors (AI use frequency, parental mediation, school support) emerged as stronger differentiators of profile membership than demographic characteristics. This pattern is consistent with social cognitive theory (Bandura, 2001), indicating that AI literacy is a malleable, context-sensitive competency shaped by reciprocal interactions among personal behaviors, environmental affordances, and cognitive processes. Importantly, the strong predictive power of school AI support, even after controlling for family SES, supports the digital inclusion perspective (Kim et al., 2021), suggesting that schools can partially compensate for home-based disadvantages.

First, AI usage frequency is the strongest predictor among all variables. Students who frequently use AI tools are significantly more likely to belong to higher-level AI literacy categories—particularly the Moderate-Stable and High-Excellence groups. This finding is consistent with previous research on university students. For example, Toker Gokce et al. (2025), using decision-tree analysis, identified AI application usage frequency as the most influential factor shaping students' AI literacy. These results collectively support the findings of Pala and Başibüyük (2020), which indicate that the frequency of technology use is directly associated with digital literacy. Together, these studies underscore the importance of sustained engagement with technological tools in deepening understanding and strengthening proficiency.

Second, family SES and parental active mediation also demonstrate significant effects. These variables draw upon the frameworks proposed by Koch et al. (2024) and Ren et al. (2022), which show that SES and parental mediation operate, respectively as outcome-related and process-related family factors influencing adolescents' digital competencies. Our findings support the relevance of these factors in shaping students' AI literacy. Moreover, compared with the study by Zhang et al. (2024), which focused on students in higher education, our work further extends this relationship to the K-12 context. The results suggest that youth who already enjoy greater social advantages—such as those from higher-SES families, and those with stronger parental mediation—also hold corresponding advantages in AI literacy. Digital inequality among adolescents has long been recognized, and existing research suggests that the traditional “digital divide” has not disappeared but rather evolved into an “intelligent divide” in the era of AI (Carter et al., 2020). Our findings offer empirical evidence for the formation of this emerging divide among primary and secondary school students. This evolution indicates that despite the growing accessibility of AI technologies, digital inequalities rooted in social backgrounds remain unmitigated.

Finally, we found that school AI support significantly distinguished students across different literacy typologies. This result is consistent with previous studies on the relationship between school ICT resources and the development of students' digital skills (Loh et al., 2025). It reinforces the perspective of school digital inclusion, positing that schools bear the responsibility of ensuring all students benefit from AI technologies (Kim et al., 2021). To build an inclusive school environment for cultivating AI literacy, schools must prioritize AI infrastructure development and teacher capacity building, adopting inclusive pedagogies that guarantee equitable learning opportunities for all students.

### 5.3 Differences in students' self-regulated learning across profiles

This study found significant differences in students' SRL across the identified latent profiles of AI literacy, with a clear gradient pattern: from the Foundational-Limited to the High-Excellence profile, students demonstrated progressively stronger performance in goal setting, goal monitoring, and performance tracking. This positive association aligns with prior research (Ji et al., 2025; Zhang and Chen, 2025); however, it is important to note that most existing studies conceptualize AI literacy as a single, homogeneous construct, thereby overlooking the potential heterogeneity within student populations. A principal contribution of this study lies in identifying four distinct latent profiles of AI literacy and illustrating how these profiles systematically correspond to differentiated levels of SRL. By adopting a person-centered approach rather than relying solely on variable-centered measures, this study unveils structural variations in students' AI literacy that deepen our understanding of how AI literacy shapes students' SRL.

These findings offer meaningful implications for the design of intelligent learning technologies. Leveraging AI and learning analytics to provide personalized support based on learners' varying AI literacy levels may lead to more accurate measurement, prediction, and enhancement of SRL processes. This has practical value for learners, educators, educational technology developers, and policymakers alike. For instance, as AI tools are increasingly integrated into SRL support systems, students' AI literacy differences should be treated as a critical design consideration rather than focusing exclusively on the functions or effectiveness of the AI tools themselves.

Building on this insight, we further propose specific pedagogical strategies for teachers to implement differentiated SRL scaffolding. Specifically, teachers should provide tailored SRL support based on students' AI literacy profiles: for Foundational-Limited and Moderate-Stable students, cultivate forethought skills; for Advanced-Developing students, strengthen self-monitoring training; for High-Excellence students, encourage peer scaffolding and AI-mediated collaborative learning. Such a human-centered approach to applying AI in K-12 education can better promote equity and effectiveness in learning (Järvelä et al., 2023).

## 6 Limitations

Several limitations should be noted. First, although the sample was large and nationally diverse within China, the findings are situated within a single cultural and educational context, which may limit cross-national generalizability. Our results are most directly applicable to K-12 students in countries with strong AI education policies and digital infrastructure (e.g., China, Singapore, South Korea), but generalization to regions with different educational systems or cultural attitudes toward AI (e.g., Europe, the United States, Africa) should be made with caution. The findings also do not automatically generalize to preschool children or university students, who differ in cognitive readiness and learning autonomy. Second, the cross-sectional design restricts causal interpretation of the relationships among AI literacy profiles, contextual factors, and SRL. Longitudinal approaches, such as latent transition analysis, are needed to examine developmental changes over time. Third, reliance on self-reported measures may introduce response bias; future studies should incorporate complementary data sources, including performance-based assessments or learning analytics.

## Data Availability

The raw data supporting the conclusions of this article will be made available by the authors, without undue reservation.
